# Tangential Biopsy Thickness versus Lesion Depth in Longitudinal Melanonychia: A Pilot Study

**DOI:** 10.1155/2012/353864

**Published:** 2012-03-14

**Authors:** Nilton Di Chiacchio, Walter Refkalefsky Loureiro, Nilceo Schwery Michalany, Felipe Veiga Kezam Gabriel

**Affiliations:** ^1^Dermatologic Clinic, Hospital do Servidor Público Municipal de São Paulo, Rua Castro Alves 131, 01532-001 São Paulo, SP, Brazil; ^2^Laboratório Paulista de Dermatologia Avenida Brigadeiro Luís Antônio 4315, Universidade Federal de São Paulo, 01401-002 São Paulo, SP, Brazil

## Abstract

Longitudinal melanonychia can be caused by melanocyte activation (hypermelanosis) or proliferation (lentigo, nevus or melanoma). Histopathologic examination is mandatory for suspicious cases of melanomas. Tangential biopsy of the matrix is an elegant technique avoiding nail plate dystrophy, but it was unknown whether the depth of the sample obtained by this method is adequate for histopathologic diagnosis. Twenty-two patients with longitudinal melanonychia striata were submitted to tangential matrix biopsies described by Haneke. The tissue was stained with hematoxylin-eosin and the specimens were measured at 3 distinct points according to the total thickness: largest (A), intermediate (B) and narrowest (C) then divided into 4 groups according to the histopathologic diagnosis (G1: hypermelanosis; G2: lentigos; G3: nevus; G4: melanoma). The lesions were measured using the same method. The mean specimen/lesion thickness measure values for each group was: G1: 0,59/0,10 mm, G2: 0,67/0,08 mm, G3: 0,52/0,05 mm, G4: 0,58/0,10 mm. The general average thickness for all the specimens/lesions was 0,59/0,08 mm. We concluded that the tangential excision, for longitudinal melanonychia, provides an adequate material for histopathological diagnosis.

## 1. Introduction

 The longitudinal melanonychia can be caused by activation (hypermelanosis) or melanocytic proliferation (lentigo, nevus, or melanoma). The diagnostic accuracy of melanomas presenting as longitudinal melanonychia is low among dermatologists [1]. Thus, biopsy of suspect cases is mandatory. Of all the differential diagnoses [2], melanoma is the more concerning [3, 4]. Among the many techniques in the literature [5–8], the tangential biopsy of the matrix [9] is an elegant alternative providing adequate samples for histopathologic analysis [10], as well as excellent cosmetic results. However, there is still question if the depth of the sample obtained by this method is adequate for histopathologic diagnosis.

 The goal of this pilot study is to demonstrate that the tangential biopsy of the matrix provides adequate specimens for the pathologist diagnosis in patients with melanonychia striata.

### 1.1. Material and Methods

**Figure 1:**
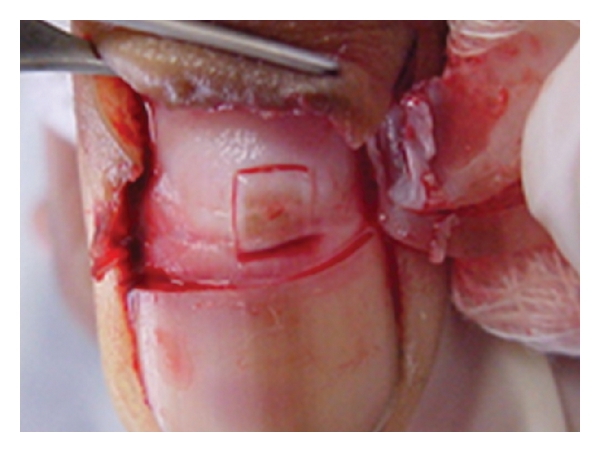
Incision around the pigmented lesion.

**Figure 2:**
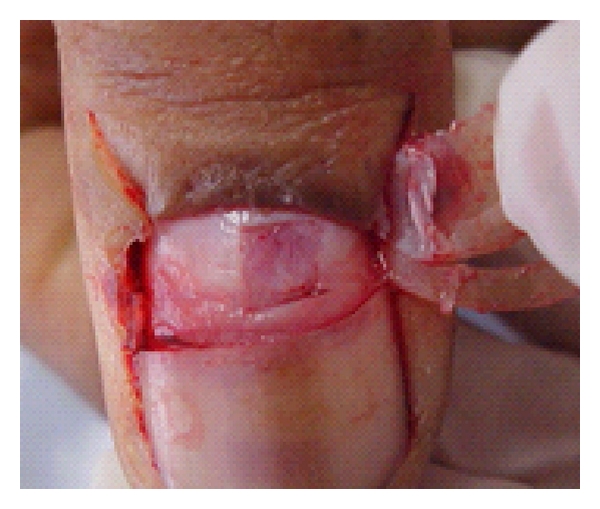
Matrix after tangential excision.

**Figure 3:**
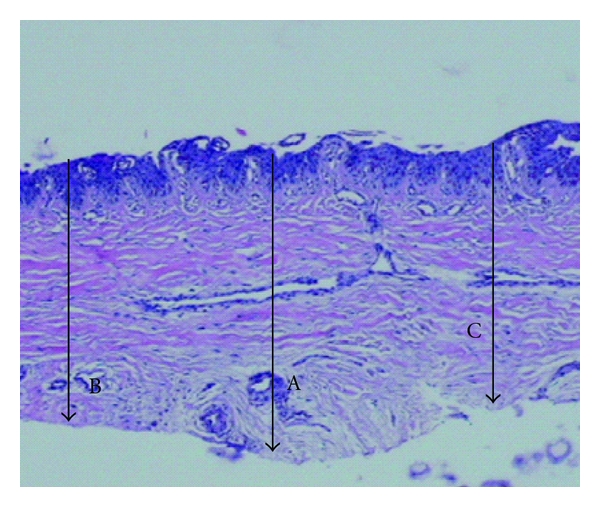
Specimen thickness measure points: largest: A, intermediate: B, and narrowest: C.

**Figure 4:**
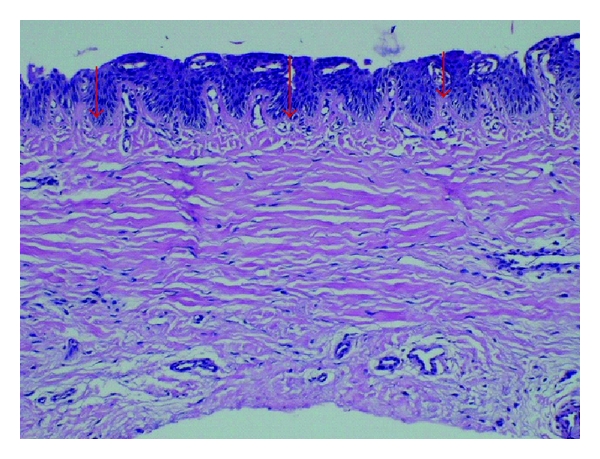
Lesion thickness measure points: largest: A, intermediate: B, and narrowest: C.

 Twenty-two cases of longitudinal melanonychia underwent matrix tangential biopsy between February 2008 and November 2010. Patients with melanonychia striata were included. Exclusion criteria were presence of Hutchinson's sing and onychodystrophy. All the biopsies were performed by the same senior dermatologic surgeon based on the technique described by Haneke and Baran [9] (Figures 1 and 2). After distal wing block of the finger, 2 oblique incisions were made at the proximal nail fold allowing to reflect it and expose the proximal nail plate. The proximal third of the nail plate is then carefully avulsed granting direct visualization of the matrix and the melanocytic lesion. Polarized dermoscopy was used during the procedure to determine the lateral margins of the lesion where gentle incisions are made. The specimen was then removed by tangential incision, placed on a piece of paper, and sent to the lab in a formalin jar. The tissue was stained with hematoxylin-eosin and analysed by the same dermatopathologist expert in histopathology of the nail apparatus. Using a microscope microruler, the specimens were measured at 3 distinct points according to the total thickness: largest: A, intermediate: B, and narrowest: C (Figure 3). The lesions depth was measured with the same method (Figure 4). The data was divided in 4 groups according to the histopathologic diagnosis: G1: hypermelanosis, G2: lentigo, G3: nevus, and G4: melanoma. The mean thickness values found in A, B, and C were calculated (MA, MB, and MC) for the specimens and lesions. The average value of the measures MA, MB, and MC (MABC) for each specimen and lesion was also calculated, as well as the general average values for all specimens (MGe) and lesions (MGl) thickness.

## 2. Results

 The mean specimen/lesion thickness measure values (MABC) for each group were: G1: 0.59/0,10 mm, G2: 0.67/0,08 mm, G3: 0.52/0,05 mm, and G4: 0.58/0,10 mm. The general average thickness for all the lesions (MGs/MGl) was 0.59/0,08 mm (Table 1).

## 3. Discussion

 The longitudinal melanonychia is described as a brown or black linear macule caused by melanocytic activation or proliferation. In melanocytic activation, the number of melanocytes is normal but with an increased pigment production. On the other hand, in the melanocytic proliferation there is an increased number of melanocytes as well as pigment production. This group includes benign hyperplasia (lentigo), nevus, and melanoma [2].

 Early diagnosis of nail apparatus melanoma remains a challenge, even among experienced dermatologists [1, 11]. The ABCDEF [12] rule is intended to facilitate the clinical detection of subungual melanoma, but it has limitations. Dermoscopy of the nail plate helps determinate if the pigment is melanic or not [2]; however, it is not as useful for proliferative melanonychia differentiation. Intraoperative dermoscopic criteria were proposed to set apart melanocytic activation from proliferation, as well as benign from malignant proliferation [13, 14]. Despite all these methods, the histopathologic exam remains the gold standard for melanonychia. Therefore, biopsy in suspected cases is mandatory.

 The histopathological alterations in hypermelanosis, lentigo, and nevus are restricted to the epidermis or upper dermis. The authors observed that when melanomas are clinically presented as longitudinal melanonychia they usually are in situ or microinvasive.

 Jellinek recently reviewed and described the biopsy techniques for melanonychia [10]. Lateral longitudinal excision and full thickness excision allow complete removal of the melanocytic lesion, but nail plate dystrophy may occur. When the 3 mm punch is used, dystrophy can also be observed, specially with children where the fingers are small. Besides, a 3 mm punch may not remove the whole lesion. All the specimens obtained by these methods is adequate for an accurate histopathological diagnosis. The tangential biopsy, described by Haneke and Baran [9], permits to completely remove the lesion without permanent dystrophy. However, there are no current studies indicating if the thickness of the excised specimens is adequate for the histopathological diagnosis.

**Figure 5:**
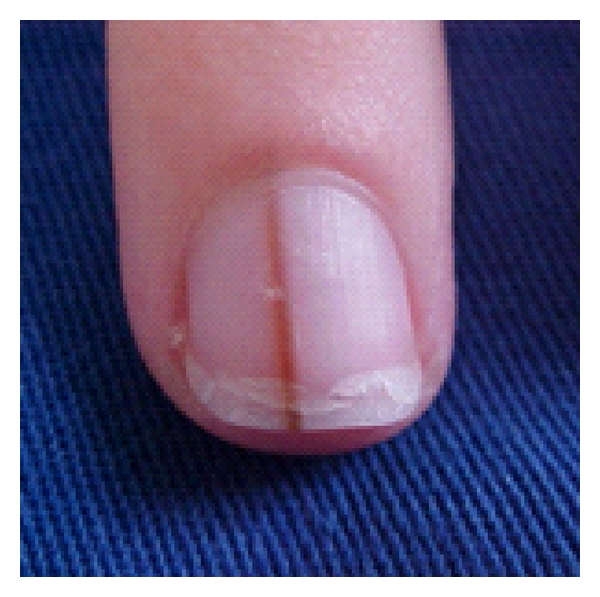
Longitudinal melanonychia.

**Figure 6:**
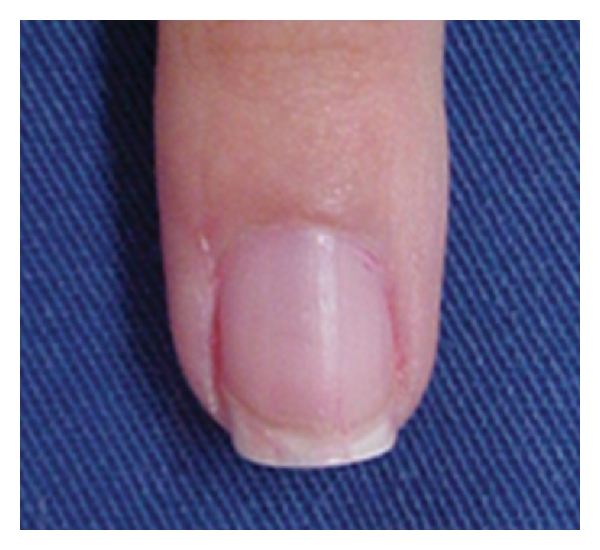
The same patient 9 months after tangential biopsy.

 In this pilot study we observed that the specimens had an average thickness of 0.59 mm, while the histopathological alterations had an average of 0.08 mm. The mean specimen depth was 7.35 times thicker than the lesion, which is very adequate for histopathological diagnosis. We also verified that the injury to the matrix by the shave biopsy is minimal and results in more aesthetic outcomes (Figures 5 and 6). It is important to keep in mind that the dermatologist, pathologist, and lab technician must be well trained and familiar with this technique.

 Although the tangential biopsy of melanocytic lesions does not provide a precise Breslow index, melanomas presenting as longitudinal melanonychia are in situ or microinvasive. Thus, the Breslow index is not measured.

 This pilot study is limited by the fact that only the depth of the lesions was addressed and it did not control the lateral aspect. No immunohistochemical analysis was realized. New studies are being conducted in this direction.

## 4. Conclusion

 Based on the measures obtained of the specimens and pigmented lesions thickness, we concluded that the tangential excision, for longitudinal melanonychia, provides an adequate material for histopathological diagnosis.

## Figures and Tables

**Table 1:** Average thickness measures.

Diagnosis	Specimens	Lesions
Hypermelanosis (G1) (*n* = 14)	MA: 0.71 mm	MA: 0.11 mm
	MB: 0.54 mm	MB: 0.10 mm
	MC: 0.53 mm	MC: 0.09 mm
	Average: **0.59 **mm	Average: **0.10 **mm

Lentigo (G2) (*n* = 1)	MA: 0.9 mm	MA: 0.1 mm
	MB: 0.6 mm	MB: 0.08 mm
	MC: 0.5 mm	MC: 0.06 mm
	MABC: **0,67 **mm	MABC: **0.08 **mm

Nevus (G3) (*n* = 6)	MA: 0.65 mm	MA: 0.07 mm
	MB: 0.47 mm	MB: 0.05 mm
	MC: 0.45 mm	MC: 0.04 mm
	MABC: **0.52 **mm	MABC: **0.05 **mm

Melanoma (G4) (*n* = 1)	MA: 0.68 mm	MA: 0.12 mm
	MB: 0.56 mm	MB: 0.10 mm
	MC: 0.51 mm	MC: 0.09 mm
	MABC: **0.58 **mm	MABC: **0.10 **mm

General average (*n* = 22)	MGs:** 0.59 **mm	MGl:** 0.08 **mm
